# Immunophenotypic Profiling of Lymphocyte Subsets in Children and Adolescents With Acute Lymphoblastic Leukaemia: Immune Dysregulation Before and After Remission

**DOI:** 10.1155/jimr/5591215

**Published:** 2026-07-02

**Authors:** Hanaa Nafady-Hego, Arwa Flemban, Saeed Kabrah, Hanan M. Abd Elmoneim, Enas Aly, Mohamed Elgendy, Talat Albukhari, Hamed Elgendy, Hams Alaa, Ruba Almaghrabi, Samar N. Ekram, Asmaa Nafady

**Affiliations:** ^1^ Microbiology and Immunology Department, Faculty of Medicine, Assiut University, Assiut, Egypt, aun.edu.eg; ^2^ Laboratory Department, Al Tahrir Medical Centre, Doha, Qatar; ^3^ Department of Pathology, Faculty of Medicine, Umm Al-Qura University, Makkah, Saudi Arabia, uqu.edu.sa; ^4^ Department of Clinical Laboratory Sciences, Faculty of Applied Medical Sciences, Umm Al-Qura University, Makkah, Saudi Arabia, uqu.edu.sa; ^5^ Department of Pathology, Faculty of Medicine, Minia University, Minia, Egypt, minia.edu.eg; ^6^ Paediatrics Department, Al Tahrir Centre, Doha, Qatar; ^7^ Paediatrics Department, Cambridge Consultant Medical Centre, Doha, Qatar; ^8^ School of Medical Sciences Health Campus, Universiti Sains Malaysia, Kubang Kerian, Kelantan, Malaysia, usm.my; ^9^ Department of Haematology and Immunology, Faculty of Medicine, Umm Al-Qura University, Makkah, Saudi Arabia, uqu.edu.sa; ^10^ Anaesthesia Department, Hamad Medical Corporation, Doha, Qatar, hamad.qa; ^11^ Weill Cornell Medical College, Doha, Qatar, cornell.edu; ^12^ Faculty of Medicine, Qatar University, Doha, Qatar, qu.edu.qa; ^13^ Anesthesia Department, Assiut University, Assiut, Egypt, aun.edu.eg; ^14^ Faculty of Medicine, Qena University, Qena, Egypt; ^15^ Department of Laboratory Medicine, Faculty of Applied Medical Sciences, Al-Baha University, Al-Baha, Saudi Arabia, bu.edu.sa; ^16^ Department of Medical Genetics, Faculty of Medicine, Umm Al-Qura University, Makkah, Saudi Arabia, uqu.edu.sa; ^17^ Department of Clinical and Chemical Pathology, Faculty of Medicine, Qena University, Qena, Egypt

**Keywords:** acute lymphoblastic leukaemia, memory Treg, naïve Treg, remission

## Abstract

**Background:**

Acute lymphoblastic leukaemia (ALL) in children is often associated with immune dysfunction, including alterations in regulatory T cells (Treg), impaired T‐cell activation, and disrupted natural killer (NK) cell function. ALL comprises immunophenotypically heterogeneous entities, primarily B‐cell ALL (B‐ALL) and T‐cell ALL (T‐ALL). However, the dynamics of Treg subsets and their relationship with broader immune alterations across treatment stages remain insufficiently characterised.

**Methods:**

In this prospective case–control study, peripheral blood samples were analysed from 31 children with newly diagnosed ALL (pre‐remission), 20 patients in remission and 24 healthy controls (HCs). Multiparameter flow cytometry was used to assess T‐cell receptor (TCR) αβ^+^/γδ^+^ T cells, CD4^+^/CD8^+^ T‐cell subsets, B cells, NK and NKT‐cell populations, CD69 activation markers, and classical and memory Treg subsets.

**Results:**

At diagnosis, patients showed significant reductions in TCR αβ^+^ and γδ^+^ T cells, CD4^+^ T‐helper cells and CD69 activation, alongside increased CD8^+^ T cells and NKT cells. Classical and memory Treg cells were significantly expanded in the pre‐remission group. Following remission, TCR expression and CD3^+^ T‐cell frequencies approached levels observed in HCs, and CD4^+^CD69^+^ activation improved. However, CD56^bright^ NK cells remained reduced, and B‐cell depletion persisted in both ALL groups.

**Conclusion:**

Paediatric ALL is characterised by marked immune dysregulation involving expanded Treg populations and impaired lymphocyte activation. Although partial immune recovery occurs after remission (AR), persistent abnormalities in NK‐cell and B‐cell compartments suggest incomplete immune reconstitution.

## 1. Introduction

Acute lymphoblastic leukaemia (ALL) represents the most prevalent form of cancer in children, accounting for ~25%–30% of paediatric malignancies and characterised by the malignant proliferation of immature lymphoid progenitors that disrupt normal haematopoiesis and immune homeostasis [1, 2]. Although the advent of risk‐adapted chemotherapy regimens has significantly improved survival outcomes, particularly in high‐income settings, treatment‐related toxicities and relapse remain significant clinical challenges [3]. These limitations have stimulated growing interest in understanding the immunological derangements associated with ALL, which may offer novel targets for more precise and less toxic therapeutic strategies.

The immune microenvironment in ALL plays a critical role in disease progression and therapy resistance. Leukaemic blasts have been shown to exploit immune checkpoints, inhibit antigen presentation, and induce T‐cell dysfunction, thereby promoting immune escape and impairing anti‐tumour responses [4]. Among adaptive immune components, CD4^+^ helper and CD8^+^ cytotoxic T cells, as well as natural killer (NK) cells from the innate immune system, are essential for leukaemic control; yet, in ALL, their functionality is often compromised due to tumour‐induced suppression [5]. In contrast, regulatory T cells (Treg), defined by CD4^+^ expression, high levels of CD25, and the transcription factor FOXP3, are frequently enriched in the tumour microenvironment and attenuate effector T‐cell and NK‐cell responses [6–8]. Elevated Treg cell frequencies have been reported in paediatric ALL, particularly at diagnosis, and their presence has been correlated with a poorer prognosis and greater immune suppression [9–11]. These observations suggest a pivotal role for Treg cells in modulating the leukaemia immune landscape, facilitating disease persistence, and impeding therapeutic efficacy.

Treg cells are not a homogeneous population but consist of phenotypically and functionally distinct subsets, including naïve (CD4^+^CD25^++^CD45RA^+^) and memory (CD4^+^CD25^+++^CD45RA^−^) cells. Naïve Treg cells, which are more prominent in early life, play a crucial role in maintaining peripheral tolerance and immune quiescence. In contrast, memory Treg cells exhibit a heightened suppressive capacity and are typically expanded in the context of chronic inflammation or malignancy [12, 13]. Evidence from haematologic malignancies suggests that memory Treg cells dominate during active disease states, contributing to resistance to immune‐mediated clearance [14, 15]. Nevertheless, the distribution and functional relevance of these Treg cell subsets in paediatric ALL, particularly across the treatment timeline from diagnosis to remission, remain inadequately defined. Moreover, other key components of immune regulation, such as NK and NKT‐cell populations, as well as early T‐cell activation markers like CD69, are understudied in this context, which limits a comprehensive understanding of the immunological alterations underpinning ALL [16].

Despite the increasing recognition of immune dysregulation in ALL, several knowledge gaps persist. Most notably, the immunophenotypic characteristics of Treg cell subsets and their interaction with effector and innate lymphocytes during different disease stages remain poorly delineated. The extent to which immune function recovers following remission, as well as the persistence of any immunological abnormalities after therapy, remains unclear. Furthermore, the potential of Treg phenotypes as prognostic markers or therapeutic targets in paediatric ALL has yet to be fully realised, as existing studies have primarily focused on adult populations or lacked detailed subset analysis [17, 18]. Addressing these gaps is essential to elucidate the immune mechanisms that support ALL progression, inform immunotherapeutic design, and reduce the need for prolonged exposure to cytotoxic agents, which are associated with long‐term toxicities in children.

ALL comprises multiple immunophenotypic and molecular subgroups, primarily B‐cell ALL (B‐ALL) and T‐cell ALL (T‐ALL), which differ in developmental origin, clinical features, and risk factors. These differences influence immune homeostasis. T‐ALL is characterised by aberrant T‐cell proliferation, whereas B‐ALL induces immune dysfunction through marrow infiltration, cytokine imbalance and therapy‐induced lymphodepletion. Thus, ALL immunological profiles must be carefully examined for disease phenotype, stage, and subtype distribution [19, 20].

In this study, we investigated the immunophenotypic landscape of peripheral blood in paediatric ALL by comparing children at diagnosis and following remission with healthy age‐matched controls (healthy controls [HCs]). Specifically, we examined the distribution of Treg cell subsets, including naïve and memory phenotypes, along with T‐cell receptor (TCR) expression, CD69 activation status, and NK/NKT‐cell profiles. Through multiparameter flow cytometry, we aimed to characterise immune alterations across disease stages, define the extent of immune reconstitution post‐treatment, and identify potential cellular targets for future immunomodulatory interventions. These findings are expected to provide a more nuanced understanding of immune dynamics in paediatric ALL and contribute to the development of novel therapeutic approaches aimed at improving immune competence and long‐term clinical outcomes.

## 2. Methodology

### 2.1. Study Design and Ethical Considerations

This prospective case–control study was conducted to evaluate the immunophenotypic profiles of lymphocyte subsets in children and adolescents with ALL, with a focus on characterising immune changes before and after remission (AR).

The study was carried out at Qena, Egypt, over 5 months from November 2022 to March 2023. Ethical approval was obtained from the Institutional Review Board of the Faculty of Medicine, Qena University (Approval Reference: SVU_MED_CCP031_4_22_11_48).

All procedures were performed in accordance with the principles outlined in the Declaration of Helsinki. Written informed consent was obtained from each participant or their legal guardian prior to enrolment.

### 2.2. Study Population

The study cohort comprised 75 children and adolescents aged 19 years or younger, stratified into three distinct groups. The first group included 31 newly diagnosed ALL patients (ALL before remission, ALL‐BR) who had not yet commenced chemotherapy at the time of enrolment. The second group consisted of 20 patients who had completed induction therapy and achieved clinical remission (ALL‐AR). A third group of 24 age‐ and sex‐matched healthy individuals served as HC. Participants in the control group were screened to ensure the absence of haematological malignancies, autoimmune diseases, chronic infections, or metabolic disorders. The management of ALL is mostly guided by the European Society for Medical Oncology (ESMO), which has established specialised protocols for children and adolescents. Treatment entails rigorous multi‐agent chemotherapy, including methotrexate (MTX), vincristine (VCR), prednisone, and cytarabine (Ara‐C), with treatment intensity modified according to risk factors and response, particularly minimal residual disease (MRD) [20]. No patients were excluded based on white blood cell (WBC) counts at diagnosis. Cases of hyperleukocytosis (>100 × 10^9^/L) were not encountered in the study cohort during the recruitment period. Patients in the ALL‐AR group were distinct individuals who had completed induction and were in complete remission, recruited independently during the study period.

Exclusion criteria for all groups included the presence of coexisting haematological conditions, such as thalassaemia or haemophilia, immunodeficiencies and any ongoing inflammatory or systemic illnesses.

### 2.3. Laboratory Assessments

All participants underwent routine haematological investigations before immunophenotyping. Complete blood counts (CBC) were performed using the Sysmex XN‐1000 Automated Haematology Analyser (Sysmex Corporation, Kobe, Japan), which provided differential WBC counts, red blood cell indices, platelet counts and reticulocyte parameters. WBC subtypes, including neutrophils, lymphocytes, monocytes, eosinophils and basophils, were quantified and verified by peripheral blood smear review when necessary. Additional routine biochemical data are provided in Table S1.

### 2.4. Sample Collection and Peripheral Blood Mononuclear Cell (PBMC) Isolation

Peripheral venous blood samples (4 mL) were collected from each subject using sterile, heparinised collection tubes. Samples were processed within 2 h of the collection to maintain cellular integrity. PBMCs were isolated using density gradient centrifugation with Histopaque‐1077 (Sigma–Aldrich, St. Louis, MO, USA), following the manufacturer’s instructions. The resulting cell pellets were washed with phosphate‐buffered saline (PBS) and prepared for immunophenotypic analysis.

### 2.5. Flow Cytometric Analysis and Antibody Panel

Immunophenotyping of lymphocyte subsets and Treg cell populations was performed using a four‐colour flow cytometric approach on a FACSCalibur flow cytometer (Becton Dickinson, San Jose, CA, USA). Fluorescence data were acquired and analysed using CellQuest software. A minimum of 40,000 lymphocyte events, gated on CD45^+^ lymphocytes and forward/side scatter characteristics, were collected per sample.

Fluorochrome‐conjugated monoclonal antibodies (mAbs) were obtained from BD Biosciences (San Diego, CA, USA) and used for surface staining. The antibody panel included markers for general lymphocyte identification (CD3, CD4, CD8 and CD19), NK and NKT‐cell characterisation (CD3, CD16 and CD56), T‐cell activation (CD69), (TCRs αβ and TCR γδ), and Treg markers (CD25 and CD45RA). Cells were incubated with the antibody cocktail for 30 min at 4°C in the dark, followed by two washes with PBS, before acquisition. Isotype‐matched control antibodies were employed to establish gating thresholds and control for non‐specific binding.

### 2.6. Immunophenotypic Definitions and Gating Strategy

Lymphocytes were identified by FSC/SSC characteristics and confirmed through CD45 expression. T cells were defined as CD3^+^ and further subtyped into CD4^+^ (T‐helper cells) and CD8^+^ (cytotoxic) populations. Additional classification included double‐positive (CD4^+^CD8^+^) and double‐negative (CD4^−^ CD8^−^) subsets. B cells were identified as CD19^+^ events within the CD45^+^ lymphocyte gate. NK cells were defined as CD3^−^ CD56^+^ and further stratified by CD56 expression intensity into CD56^bright^ and CD56^dim^ subsets. NKT cells were defined as CD3^+^CD56^+^.

TCR expression was assessed by identifying TCR αβ^+^ and TCR γδ^+^ cells within the CD3^+^ population. The activation status of lymphocyte subsets was determined by evaluating the CD69 expression on CD3^+^, CD4^+^ and CD8^+^ cells.

Treg cells were analysed within the CD3^+^CD4^+^ population based on differential CD25 expression. CD25‐high cells (fluorescence intensity > 10^2^) were categorised as classical Treg cells. Additional CD25 expression strata (CD25^+^, CD25^++^ and CD25^−^) were quantified to provide a complete profile of activation and regulatory states. For further delineation of Treg cell phenotypes, co‐expression of CD25 and CD45RA was used to classify naïve (CD25^++^CD45RA^+^) and memory (CD25^+++^CD45RA^−^) Treg cell subsets, along with four additional intermediate populations.

The full sequential flow cytometry gating strategy, including lymphocyte selection by FSC/SSC, confirmation within the CD45^+^ gate, and downstream identification of T‐cell, B‐cell, NK/NKT‐cell, TCR, CD69 and CD25/CD45RA‐defined Treg subsets, is provided in Figure S1.

### 2.7. Statistical Analysis

Statistical analyses were performed using SPSS Version 30.0 (IBM Corp., Armonk, NY, USA). The Shapiro–Wilk test was used to assess the normality of the distribution for all continuous variables. As most data were non‐parametric, group comparisons were conducted using the Mann–Whitney *U* test, and results were expressed as medians with interquartile ranges (IQR). Categorical variables were analysed using the chi‐square test. Statistical significance was defined as a two‐tailed *p*‐value of less than 0.05.

## 3. Results

### 3.1. Participants’ Demographic and Haematological Characteristics

The study population consisted of 75 children and adolescents divided into three groups: 31 patients with newly diagnosed ALL (ALL‐BR), 20 patients in remission after induction therapy (ALL‐AR), and 24 HCs. The demographic and baseline haematological characteristics of the study population are summarised in Table 1. The median age was comparable across the groups, although minor statistical differences were observed between the patient groups and controls. No significant differences were identified in the gender distribution or body mass index.

**Table 1:** Demographic and haematological characteristics of paediatric patients with acute lymphoblastic leukaemia (ALL) and healthy controls (HC).

Baseline demographic characteristics of study participants						
Variables	ALL‐BR (*n* = 31)	ALL‐AR (*n* = 20)	HC (*n* = 24)	*p*1 value ALL‐BR vs. ALL‐AR	*p*2 value ALL‐BR vs. HC	*p*3 value ALL‐AR vs. HC
Age (years), median (IQR)	15.3 (14.5 –16.0)	16 (14.3–16.0)	15.0 (14.0–16.0)	0.635	0.008	0.005
Gender (male/female)	9/22	10/10	8/16	0.322	—	—
BMI, median (IQR)	24.7 (18.0–28.7)	24.7 (18.9–28.7)	—	0.917	—	—
ALL subtype, *n* (%)						
B‐ALL	21 (41.2%)	13 (25.5%)	—	—	—	—
T‐ALL	10 (19.6%)	7 (13.7%)	—	—	—	—
**Haematological characteristics**						
WBC (×10^9^/L)	5.7 (3.3–8.8)	7.2 (4.7–7.5)	8.8 (7.7–10.8)	0.439	<0.001	0.005
Lymphocytes (%)	30.1 (17.8–38.0)	20.0 (16.5–42.9)	25.7 (17.1–47.3)	0.474	0.610	0.210
Lymphocyte count (×10^9^/L)	1.9 (1.3–3.5)	1.2 (0.93–2.3)	2.4 (2.0–3.3)	0.153	0.002	0.120
LNR	1.1 (0.5–3.2)	0.6 (0.2–1.5)	0.41 (0.22–1.13)	0.058	0.005	0.417
CD4/neutrophil ratio	0.3 (0.1–0.8)	0.2 (0.1–0.5)	0.17 (0.11–0.31)	0.192	0.144	0.854
CD8/neutrophil ratio	0.4 (0.1–0.7)	0.2 (0.1–0.4)	0.12 (0.06–0.22)	0.264	0.007	0.132
CD4/CD8 ratio	1.2 (0.9–1.5)	1.0 (0.7–1.4)	1.5 (1.3–1.9)	0.581	0.008	0.003
PLR	30 (9–117)	246 (137–1287)	170 (76–218)	<0.001	0.011	0.147
Platelet/CD4 ratio	176 (57–482)	686 (444–686)	410 (297–536)	<0.001	0.067	0.058
Platelet/CD8 ratio	168 (46–567)	796 (325–7018)	623 (445–834)	<0.001	.002	0.871

*Note*: Statistical comparisons: *p*1, comparison between ALL‐BR and ALL‐AR; *p*2, comparison between ALL‐BR and HC; *p*3, comparison between ALL‐AR and HC. Statistical analyses were performed using the Mann–Whitney *U* test for continuous variables and the chi‐square test for categorical variables. A *p* value < 0.05 was considered statistically significant. Data are presented as median (interquartile range, IQR) for continuous variables and number (*n*) for categorical variables. Study groups included ALL‐BR (*n* = 31), ALL‐AR (*n* = 20) and HC (*n* = 24).

Abbreviations: ALL‐AR, acute lymphoblastic leukaemia after remission; ALL‐BR, acute lymphoblastic leukaemia before remission; BMI, body mass index; HC, healthy controls; HCT, haematocrit; INR, international normalised ratio; LNR, lymphocyte‐to‐neutrophil ratio; MCH, mean corpuscular haemoglobin; MCHC, mean corpuscular haemoglobin concentration; MCV, mean corpuscular volume; PLR, platelet‐to‐lymphocyte ratio; RBC, red blood cells; WBC, white blood cells.

Haematological parameters demonstrated the expected abnormalities associated with active leukaemia. Patients in the ALL‐BR group showed significantly lower haemoglobin levels, haematocrit values and platelet counts compared with the remission and control groups, reflecting bone marrow suppression at diagnosis. WBC and neutrophil counts were also significantly altered in ALL patients compared with HC. Several haematological ratios derived from these parameters, including the lymphocyte‐to‐neutrophil ratio and platelet‐based indices, differed significantly between groups.

Among the patients with ALL, 34 (67%) had B‐ALL, and 17 (33%) had T‐ALL. The distribution of ALL subtypes is summarised in Table 1, while detailed immunophenotypic comparisons between B‐ALL and T‐ALL patients before and AR are presented in Table 2.

**Table 2:** Immunophenotypic characteristics of lymphocyte subsets in B‐cell acute lymphoblastic leukaemia (B‐ALL) and T‐cell acute lymphoblastic leukaemia (T‐ALL) patients before remission (BR) and after remission (AR).

Parameters	B‐ALL (*n* = 34)		*p* values	T‐ALL (*n* = 17)		*p* values
%	B‐ALL‐BR (*n* = 21, 62%)	B‐ALL‐AR (*n* = 13, 38%)	—	T‐ALL‐BR (*n* = 10, 59%)	T‐ALL‐AR (*n* = 7, 41%)	
TCR αβ^+^	23.7 (11.6–44.7)	51.2 (33.5–63.3)	**0.03**	19.4 (10–44.8)	41.5 (34.6–52.9)	0.08
TCR γδ^+^	1 (0.6–2.5)	3.6 (2.7–5)	**0.006**	2.1 (1.3–3.2)	4.2 (3.2–4.7)	**0.01**
T cells (CD3^+^)	66 (45.3–77.4)	81.2 (72.8–85.8)	**0.05**	88.8 (41.6–91.7)	77.7 (72.1–84.5)	0.6
B cells (CD19^+^)	10.9 (4.1–17.3)	1.3 (0.9–11.1)	**0.02**	4.9 (2.6–11.1)	8.5 (0.9–11.3)	0.6
NKT cells (CD3^+^ CD56^+^)	11.2 (7.6–14.4)	9.2 (4.6–13.7)	0.33	9.1 (8.2–22.1)	11.4 (9.8–13.5)	0.55
NK cells (CD3^−^ CD56^+^)	0.4 (0.2–0.9)	0.1 (0.1–1)	0.17	0.1 (0.1–0.5)	1.3 (0.1–2.6)	**0.04**
CD56^bright^ NK cells (CD3^−^ CD56^++^)	10 (7–14.6)	5.5 (2.2–10.7)	**0.05**	2.6 (1.7–8.8)	10.3 (6.4–13.2)	0.12
T‐helper cells (CD4^+^ CD8^−^)	51 (43–58)	44.7 (40.2–52.6)	0.19	44.4 (32–46.5)	45.3 (37.3–52.5)	0.64
Cytotoxic T cells (CD8^+^ CD4^−^)	40.7 (36.8–44.2)	45.1 (35.4–52.9)	0.74	48 (37.8–60.7)	45.9 (38–53)	1
Double‐positive T cells (CD4^+^ CD8^+^)	1.1 (0.2–2.2)	0.8 (0.4–1.3)	0.35	0.5 (0.4–1)	1.1 (0–1.3)	0.64
Double‐negative T cells (CD4^−^ CD8^−^)	5.3 (2.4–11.4)	8.2 (4.1–10.4)	0.63	11.6 (3.7–14.1)	8.6 (7.1–9.8)	0.64
Classical Treg (CD25^+++^)	5.5 (3.4–7.3)	3.5 (2–6)	0.1	3.3 (2.5–5.8)	2.2 (1.4–2.8)	**0.04**
Intermediate Treg (CD25^++^)	6.2 (2.9–9)	8.5 (6.3–11.5)	0.18	5.1 (4.6–11.4)	6.3 (3.3–8.5)	0.81
Low‐expression Treg (CD25^+^)	13 (6.6–20.6)	13.8 (10.7–16.8)	0.5	14.1 (10.2–30.8)	14.8 (11.7–19.3)	0.72
Non‐Treg (CD25^−^)	20.5 (10.1–24.4)	16.2 (12.9–19.2)	0.41	15.8 (11.7–34)	20.4 (12.2–24.4)	0.72
Naïve Treg (CD25^++^ CD45RA^+^)	3.3 (0.9–8.2)	2 (1–4.2)	0.36	1.2 (1.1–2.5)	4.3 (2.9–6.1)	**0.01**
Memory Treg (CD25^+++^ CD45RA^−^)	5.8 (3.5–8.2)	6.2 (4.5–10.7)	0.6	10.2 (5–13.4)	3.8 (0.9–10.2)	1
Intermediate Treg (CD25^++^ CD45RA^−^)	14.2 (12.4–17.9)	9 (7.3–16.7)	0.09	10.2 (5–13.4)	7.1 (5.8–9.3)	0.6
Low‐expression Tregs (CD25^+^ CD45RA^−^)	21.2 (14.5–28.6)	28.9 (11.8–38.2)	0.17	29.1 (25.9–30.6)	19.7 (16.5–29.6)	0.04
Non‐Treg (CD25^−^ CD45RA^−^)	16.1 (13.8–19.4)	24.3 (21.4–29.2)	**0.001**	17.3 (15.4–38.3)	17.2 (4.9–28.7)	0.15
Non‐Treg (CD25^−^ CD45RA^+^)	13 (7.2–22.3)	9.1 (2.7–18.1)	0.25	8.9 (1.9–13.4)	14.8 (8.3–51)	0.12
CD3^+^ CD69^+^	16.7 (8.5–42.1)	44.1 (29.5–56.3)	**0.03**	20.8 (10–40.7)	37.8 (30.2–50.7)	**0.04**
CD4^+^ CD69^+^	41.5 (31–46.5)	37.5 (29.5–42.8)	0.16	32.7 (25–41.6)	41.4 (25.9–43.9)	**0.04**
CD8^+^ CD69^+^	21.1 (12.8–26.8)	24.8 (10.6–37.2)	**0.03**	39.6 (20.6–51.9)	23 (15.8–24)	0.09

*Note*: Values are presented as median (interquartile range, IQR). Statistical significance was defined as *p* < 0.05. Statistical comparisons: *p* values indicate comparisons between BR and AR within each disease subtype (B‐ALL or T‐ALL) using the Mann–Whitney *U* test. NK, natural killer cells; NKT, natural killer T cells; Treg, regulatory T cells. Bold values indicate statistically significant differences (*p* < 0.05).

Abbreviations: AR, after remission; B‐ALL, B‐cell acute lymphoblastic leukaemia; BR, before remission; T‐ALL, T‐cell acute lymphoblastic leukaemia; TCR, T‐cell receptor.

### 3.2. Immunophenotypic Alterations in Peripheral Blood Lymphocyte Subsets in Paediatric ALL Before and AR

**Figure 1:**
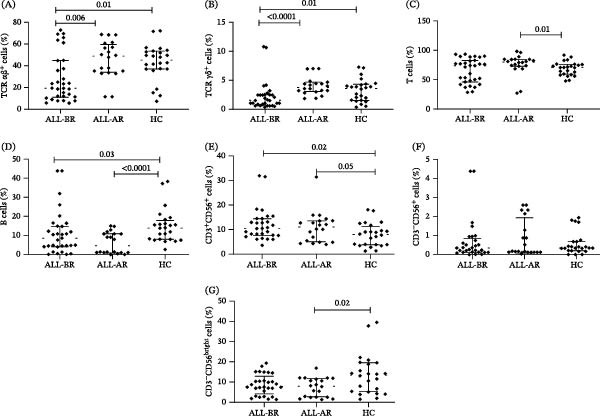
Differential expression of T‐cell receptor (TCR), lymphocyte, NK and NKT‐cell subsets in paediatric acute lymphoblastic leukaemia and healthy controls. Flow cytometric analysis of immune cell subsets in peripheral blood mononuclear cells (PBMCs) obtained from paediatric patients with acute lymphoblastic leukaemia before remission (ALL‐BR, *n* = 31), after remission (ALL‐AR, *n* = 20) and healthy controls (HC, *n* = 24). Data are presented as median percentages with interquartile range (IQR) within CD45^+^ lymphocyte‐gated populations. T‐cell receptor subsets: (A) TCR αβ^+^ T cells and (B) TCR γδ^+^ T cells, both significantly reduced in ALL‐BR compared with HC and ALL‐AR. Lymphocyte subsets: (C) total T cells (CD3^+^), significantly elevated in ALL‐AR compared with HC (ALL‐AR vs. HC, *p* = 0.01), while no significant differences were observed between ALL‐BR and HC (*p* = 0.87) or between ALL‐BR and ALL‐AR (*p* = 0.13). (D) B cells (CD19^+^), significantly reduced in both ALL‐BR (*p* = 0.03 vs. HC) and ALL‐AR (*p* < 0.0001 vs. HC), with no significant difference between ALL‐BR and ALL‐AR (*p* = 0.10). NK and NKT cell subsets: (E) NKT cells (CD3^+^CD56^+^), increased in both ALL‐BR and ALL‐AR groups. (F) Total NK cells (CD3^−^CD56^+^), showing no significant differences among groups. (G) CD56^bright^ NK cells (CD3^−^CD56^bright^), significantly reduced in ALL‐AR compared with HC. Statistical comparisons were performed using the Mann–Whitney *U* test, and *p* < 0.05 was considered statistically significant. Each data point represents an independent patient sample. Flow cytometric analysis was performed once per sample, and a minimum of 40,000 CD45^+^ lymphocyte events were acquired for analysis. ALL‐AR, acute lymphoblastic leukaemia after remission; ALL‐BR, acute lymphoblastic leukaemia before remission; HC, healthy controls; IQR, interquartile range; NK, natural killer cells; NKT, natural killer T cells; TCR, T‐cell receptor.

Flow cytometric analysis (Figure 1A,B) revealed a marked suppression in the expression of TCR subsets among ALL‐BR. The frequency of TCR αβ^+^ T cells within the CD45^+^ lymphocyte gate were significantly reduced in ALL‐BR patients compared to both HC and ALL‐AR (*p* = 0.01 and *p* = 0.006, respectively). Similarly, TCR γδ^+^ T cells were significantly less abundant in the ALL‐BR group than in the HC and ALL‐AR groups (*p* = 0.01 and *p* < 0.0001, respectively). In contrast, the ALL‐AR group displayed restoration of both TCR αβ^+^ and γδ^+^ subsets to levels comparable with those of HC, suggesting immunological reconstitution following chemotherapy‐induced remission.

The percentage of total T‐lymphocytes (Figure 1C), defined as CD3^+^ cells, was significantly higher in the ALL‐AR group when compared to HC (*p* = 0.01), while no statistically significant difference was observed between ALL‐BR and HC (*p* = 0.87). These findings suggest enhanced peripheral T‐cell reconstitution following remission. Conversely, B‐cell frequencies, identified as CD19^+^ cells, were significantly decreased in both ALL‐BR and ALL‐AR patients relative to HC (*p* = 0.03 and *p* < 0.0001, respectively). Although B‐cell depletion was more pronounced in the post‐remission phase, the difference between ALL‐BR and ALL‐AR did not reach statistical significance (*p* = 0.10), indicating persistent B‐cell suppression post‐therapy (Figure 1D).

Further characterisation of innate lymphoid compartments (Figure 1E–F) demonstrated alterations in NK and NKT‐cell frequencies. The proportion of NKT cells, defined as CD3^+^CD56^+^, was elevated in both ALL‐BR and ALL‐AR groups compared to HC, consistent with a compensatory or reactive expansion in response to disease or treatment. Although this increase did not achieve statistical significance between patient groups, the trend suggests a disease‐associated perturbation of NKT homeostasis. Total NK cells (CD3^−^ CD56^+^) showed no statistically significant variation across the three groups, implying relative preservation of this cytotoxic lymphocyte population during disease and remission phases. However, a distinct subset of NK cells, CD56^bright^ NK cells (CD3^−^ CD56^++^), was significantly reduced in ALL‐AR patients compared to HC (*p* = 0.02). This subset is typically associated with immunoregulatory and cytokine‐producing functions, and its depletion in the post‐remission state may reflect the lingering effects of chemotherapy or altered NK‐cell maturation and differentiation pathways.

### 3.3. CD4^+^ and CD8^+^ T‐Cell Subset Distribution

**Figure 2:**
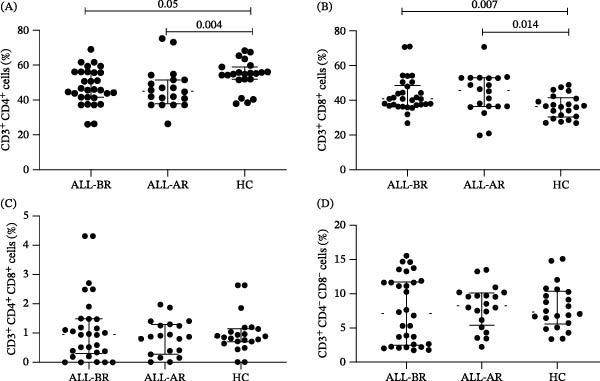
Altered frequencies of CD4^+^ and CD8^+^ T‐cell subsets in paediatric acute lymphoblastic leukaemia. Flow cytometric analysis of T‐cell subsets within CD45^+^ CD3^+^ lymphocyte‐gated peripheral blood mononuclear cells (PBMCs) obtained from paediatric patients with acute lymphoblastic leukaemia before remission (ALL‐BR, *n* = 31), after remission (ALL‐AR, *n* = 20) and healthy controls (HC, *n* = 24). Data are presented as median percentages with interquartile range (IQR). (A) T‐helper cells (CD4^+^ CD8^−^) were significantly reduced in ALL‐BR compared with HC. (B) Cytotoxic T cells (CD8^+^ CD4^−^) were significantly increased in ALL‐BR compared with HC. (C) Double‐positive T cells (CD4^+^ CD8^+^) showed no significant differences among groups. (D) Double‐negative T cells (CD4^−^ CD8^−^) also showed no significant differences among groups. Statistical comparisons between groups were performed using the Mann–Whitney *U* test, and *p* < 0.05 was considered statistically significant. Each data point represents an independent patient sample. Flow cytometry analysis was performed once per sample, and a minimum of 40,000 CD45^+^ lymphocyte events were acquired for analysis. ALL‐AR, acute lymphoblastic leukaemia after remission; ALL‐BR, acute lymphoblastic leukaemia before remission; HC, healthy controls; IQR, interquartile range.

Quantitative analysis of T‐cell subsets within the CD45^+^CD3^+^ lymphocyte population (Figure 2) revealed significant alterations in both helper and cytotoxic T‐cell frequencies in children with ALL, particularly during the pre‐remission phase. The proportion of CD4^+^CD8^−^ T‐helper cells was markedly reduced in the ALL‐BR group compared to HC (*p* = 0.004), indicating disease‐associated depletion of this immunoregulatory subset. The ALL‐AR group exhibited intermediate levels of CD4^+^ T cells, which were significantly higher than those in ALL‐BR (*p* = 0.05) and remained lower than those in HC, suggesting partial reconstitution of helper T‐cell numbers following remission. In contrast, CD8^+^CD4^−^ cytotoxic T cells were significantly increased in the ALL‐BR group compared to HC (*p* = 0.007), consistent with a shift towards cytotoxic T‐cell predominance in active disease. This elevation remained evident, although less pronounced, in the ALL‐AR group, which also showed significantly higher CD8^+^ T‐cell frequencies compared to the control group (*p* = 0.014). These findings suggest a persistent cytotoxic skewing of the T‐cell compartment, potentially reflecting the host’s immune response to leukaemia cells or chemotherapy‐induced immune modulation. No statistically significant differences were observed in the frequencies of double‐positive (CD4^+^CD8^+^) or double‐negative (CD4^−^ CD8^−^) T‐cell populations across the three study groups. The distribution of these minor subsets remained relatively stable, indicating that the most profound immunological shifts in paediatric ALL occur within the conventional single‐positive CD4^+^ and CD8^+^ T‐cell compartments.

### 3.4. CD25‐Defined Treg Subsets

**Figure 3:**
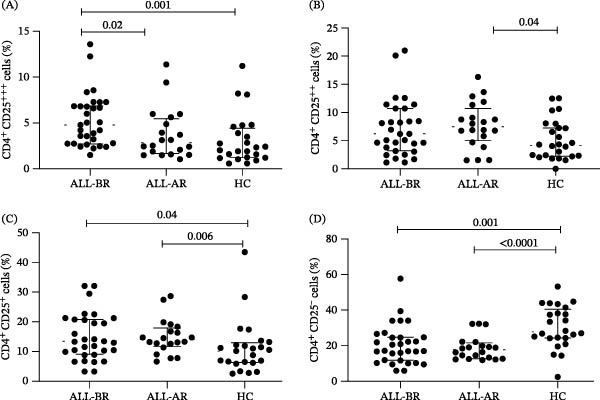
Differential expression of CD25‐defined regulatory T‐cell subsets in paediatric acute lymphoblastic leukaemia. Flow cytometric analysis of CD25 expression on CD4^+^ T cells within CD45^+^ lymphocyte‐gated peripheral blood mononuclear cells (PBMCs) obtained from paediatric patients with acute lymphoblastic leukaemia before remission (ALL‐BR, *n* = 31), after remission (ALL‐AR, *n* = 20) and healthy controls (HC, *n* = 24). Data are presented as median percentages with interquartile range (IQR) and stratified into four subpopulations according to CD25 expression intensity, which has been previously correlated with FOXP3 expression. (A) Classical regulatory T cells (CD25^+++^) were significantly expanded in ALL‐BR compared with HC and ALL‐AR. (B) Intermediate regulatory T cells (CD25^++^) showed a moderate increase in ALL‐BR. (C) Low‐expression regulatory T cells (CD25^+^) showed no significant differences among groups. (D) Non‐regulatory CD4^+^ T cells (CD25^−^) were significantly reduced in both ALL‐BR and ALL‐AR compared with HC. Statistical comparisons between groups were performed using the Mann–Whitney *U* test, and *p* < 0.05 was considered statistically significant. Each data point represents an independent patient sample. Flow cytometry analysis was performed once per sample, and a minimum of 40,000 CD45^+^ lymphocyte events were acquired for analysis. ALL‐AR, acute lymphoblastic leukaemia after remission; ALL‐BR, acute lymphoblastic leukaemia before remission; HC, healthy controls; IQR, interquartile range; Treg, regulatory T cell.

The analysis of CD4^+^ T cells, based on the surface expression of CD25 (Figure 3), revealed distinct alterations in Treg subsets among children with ALL. Classical Treg, defined as CD4^+^CD25^+++^, were significantly expanded in ALL‐BR compared to both HC and ALL‐AR, with *p*‐values of 0.001 and 0.02, respectively. This finding reflects a pronounced enrichment of highly suppressive Treg cell populations during active disease. Intermediate Treg cells (CD4^+^CD25^++^) also demonstrated a modest elevation in frequency in the ALL‐BR group relative to that in HC, although the difference did not reach statistical significance when compared to ALL‐AR. CD4^+^CD25^+^ cells, representing low‐expression Treg cells or potentially activated non‐regulatory cells, showed no significant variation across the three groups, indicating that the observed regulatory skewing was specific to the high and intermediate CD25‐expressing populations. In contrast, the proportion of CD25^−^ CD4^+^ cells, representing non‐regulatory or naïve helper T cells, was significantly reduced in both ALL‐BR and ALL‐AR groups when compared with HC (*p* = 0.001 and *p* < 0.0001, respectively). This reduction suggests a contraction of the conventional CD4^+^ T‐cell pool in favour of regulatory phenotypes during active disease and its persistence, albeit to a lesser extent, following remission. These observations provide further evidence of the immunosuppressive environment characteristic of ALL, implying that Treg cell expansion may contribute to immune evasion mechanisms in the leukaemia setting. Moreover, the persistence of altered Treg cell proportions AR suggests that immune reconstitution may remain incomplete despite clinical recovery.

### 3.5. CD25 and CD45RA Co‐Defined Treg Subsets

**Figure 4:**
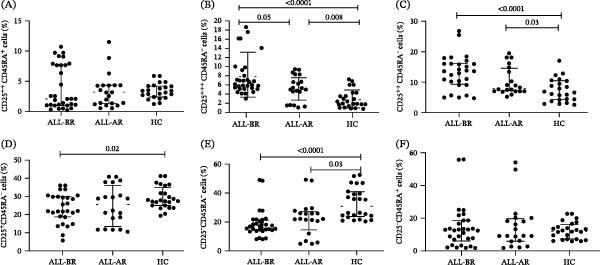
Differential distribution of CD25‐ and CD45RA‐defined regulatory T‐cell subsets in paediatric acute lymphoblastic leukaemia. Flow cytometric analysis of CD25 and CD45RA co‐expression on CD3^+^ CD4^+^ T cells within CD45^+^ lymphocyte‐gated peripheral blood mononuclear cells (PBMCs) obtained from paediatric patients with acute lymphoblastic leukaemia before remission (ALL‐BR, *n* = 31), after remission (ALL‐AR, *n* = 20) and healthy controls (HC, *n* = 24). Data are presented as median percentages with interquartile range (IQR) and stratified into six regulatory T‐cell (Treg) subsets based on CD25 and CD45RA expression, which have been previously associated with distinct FOXP3 expression patterns. (A) Naïve Treg cells (CD25^++^ CD45RA^+^) showed no significant differences among groups. (B) Memory Treg cells (CD25^+++^ CD45RA^−^) were significantly expanded in ALL‐BR compared with HC and ALL‐AR. (C) Intermediate Treg cells (CD25^++^ CD45RA^−^) were significantly increased in ALL‐BR compared with HC. (D) Low‐expression Treg cells (CD25^+^ CD45RA^−^) showed no significant differences among groups. (E) Non‐Treg cells (CD25^−^ CD45RA^−^) were significantly reduced in ALL‐BR compared with HC. (F) Non‐Treg cells (CD25^−^CD45RA^+^) showed no significant differences among groups. Statistical comparisons between groups were performed using the Mann–Whitney *U* test, and *p* < 0.05 was considered statistically significant. Each data point represents an independent patient sample. Flow cytometry analysis was performed once per sample, and a minimum of 40,000 CD45^+^ lymphocyte events were acquired for analysis. ALL‐AR, acute lymphoblastic leukaemia after remission; ALL‐BR, acute lymphoblastic leukaemia before remission; HC, healthy controls; IQR, interquartile range; Treg, regulatory T cell.

The co‐expression of CD25 and CD45RA on CD3^+^CD4^+^ lymphocytes was used to further delineate Treg cell phenotypes and assess shifts in the balance between naïve and memory Treg cell populations in children with ALL (Figure 4). The frequency of memory Treg cells (CD25^+++^ CD45RA^−^), considered the most suppressive subset, was significantly elevated in the ALL‐BR group compared to both HC and patients in remission (*p* < 0.0001 and *p* = 0.05, respectively). This expansion strongly suggests the accumulation of terminally differentiated Treg cells in active disease, likely contributing to the immune suppression associated with the progression of ALL. Intermediate Treg cells, defined as CD25^++^ CD45RA^−^, were also significantly increased in the ALL‐BR group relative to HC (*p* < 0.0001), further supporting a disease‐associated shift towards a memory‐like and potentially more immunosuppressive phenotype. In contrast, the frequency of naïve Treg cells (CD25^++^ CD45RA^+^) did not differ significantly across the study groups, indicating that the thymic output or peripheral maintenance of this subset may remain relatively unaffected in the context of ALL or its treatment. Low‐expression Treg cells (CD25^+^ CD45RA^−^) were significantly increased in the ALL‐BR group relative to HC (*p* = 0.02) while resting non‐Treg cells (CD25^−^ CD45RA^+^) showed no significant variation among ALL‐BR, ALL‐AR, and HC, suggesting a degree of immunological stability in these populations. However, the population of CD25^−^ CD45RA^−^ cells, typically considered non‐regulatory effector or transitioning CD4^+^ cells, was significantly reduced in ALL‐BR compared with HC (*p* < 0.0001) and remained lower in the remission group (*p* = 0.03), implying persistent contraction of this subset following chemotherapy. Altogether, these findings highlight a disease‐specific expansion of functionally suppressive memory Treg cell subsets in untreated ALL, with only partial resolution following induction therapy. The persistence of altered Treg cell homeostasis may have implications for immune reconstitution, disease surveillance, and the risk of long‐term relapse.

### 3.6. CD69 Expression on T‐Cell Subsets

**Figure 5:**
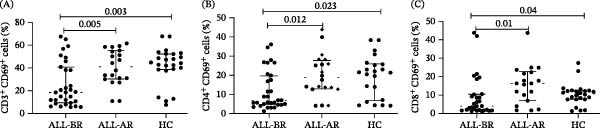
Altered expression of the CD69 activation marker in T‐cell subsets in paediatric acute lymphoblastic leukaemia. Flow cytometric analysis of CD69 expression, an early activation marker, on T‐cell subsets within CD45^+^ lymphocyte‐gated peripheral blood mononuclear cells (PBMCs) obtained from paediatric patients with acute lymphoblastic leukaemia before remission (ALL‐BR, *n* = 31), after remission (ALL‐AR, *n* = 20) and healthy controls (HC, *n* = 24). Data are presented as median percentages with interquartile range (IQR). (A) Total T cells (CD3^+^CD69^+^) were significantly reduced in ALL‐BR compared with HC and ALL‐AR. (B) T‐helper cells (CD4^+^CD69^+^) were significantly decreased in ALL‐BR but increased in ALL‐AR compared with HC. (C) Cytotoxic T cells (CD8^+^CD69^+^) were significantly increased in ALL‐BR compared with HC. Statistical comparisons between groups were performed using the Mann–Whitney *U* test, and *p* < 0.05 was considered statistically significant. Each data point represents an independent patient sample. Flow cytometry analysis was performed once per sample, and a minimum of 40,000 CD45^+^ lymphocyte events were acquired for analysis. ALL‐AR, acute lymphoblastic leukaemia after remission; ALL‐BR, acute lymphoblastic leukaemia before remission; HC, healthy controls; IQR, interquartile range.

The expression of CD69, an early activation marker, was assessed across CD3^+^, CD4^+^ and CD8^+^ T‐cell subsets to evaluate the functional activation status of lymphocytes in paediatric ALL (Figure 5). CD69^+^ expression on total T cells (CD3^+^CD69^+^) was significantly reduced in the ALL‐BR group compared to both HC (*p* = 0.005) and the ALL‐AR group (*p* = 0.003), indicating impaired early T‐cell activation during active disease. In contrast, levels in the ALL‐AR group were restored to those observed in HC, suggesting a partial recovery of activation capacity following remission. When examined within the CD4^+^ T‐cell subset, CD69^+^ expression was likewise diminished in ALL‐BR patients, with a significant difference compared to HC (*p* = 0.012). Interestingly, CD4^+^ CD69^+^ frequencies were elevated in the ALL‐AR group relative to both ALL‐BR and HC (*p* = 0.023), reflecting a rebound in helper T‐cell activation following treatment and immune reconstitution. By contrast, CD69^+^ expression on CD8^+^ T cells was significantly increased in the ALL‐BR group compared to the HC (*p* = 0.01), suggesting a selective activation of cytotoxic T cells during the active disease phase. However, this elevation was not sustained in the remission group, where levels fell significantly compared to ALL‐BR (*p* = 0.04) and were closer to those observed in HC. These findings collectively indicate that T‐cell activation in paediatric ALL is profoundly altered in a subset‐specific manner. While helper T cells demonstrate suppressed activation in active disease, with enhanced recovery post‐remission, cytotoxic T cells appear hyperactivated during active ALL, potentially reflecting an attempted immune response against malignant cells. The restoration of CD69 expression profiles AR underscores the dynamic nature of T‐cell functional status in response to the disease burden and therapy.

As shown in Table 2, exploratory subtype‐stratified analyses identified differences in immunophenotypic profiles between patients with B‐ALL and T‐ALL BR and AR. Within the B‐ALL cohort (*n* = 34), 21 patients (61.8%) were classified as B‐ALL‐BR and 13 (38.2%) as B‐ALL‐AR, whereas the T‐ALL cohort included 10 patients (58.8%) in the T‐ALL‐BR group and 7 patients (41.2%) in the T‐ALL‐AR group. Within the B‐ALL cohort, 21 patients (61.8%) were classified as B‐ALL‐BR and 13 (38.2%) as B‐ALL‐AR, whereas the T‐ALL cohort included 10 patients (58.8%) in the T‐ALL‐BR group and 7 patients (41.2%) in the T‐ALL‐AR group.

The frequency of TCR αβ^+^ T cells were significantly lower in the B‐ALL‐BR group compared with the B‐ALL‐AR group (*p* = 0.03). A similar pattern was observed for TCR γδ^+^ T cells, which were significantly reduced in the B‐ALL‐BR group relative to the remission group (*p* = 0.006). In contrast, although TCR αβ^+^ T‐cell frequencies tended to be lower in the T‐ALL‐BR group than in the T‐ALL‐AR group, this difference did not reach statistical significance (*p* = 0.08). However, TCR γδ^+^ T cells were significantly reduced in the T‐ALL‐BR group compared with the T‐ALL‐AR group (*p* = 0.01).

Regarding lymphocyte subsets, the proportion of total T cells (CD3^+^) was significantly higher in the B‐ALL‐AR group than in the B‐ALL‐BR group (*p* = 0.05), whereas the percentage of B cells (CD19^+^) was significantly lower in B‐ALL‐AR patients (*p* = 0.02). In the T‐ALL cohort, NK‐cell frequencies (CD3^−^ CD56^+^) were significantly higher in the T‐ALL‐AR group than in the T‐ALL‐BR group (*p* = 0.04). Conversely, CD56^bright^ NK cells were significantly reduced in the B‐ALL‐AR group compared with the B‐ALL‐BR group (*p* = 0.05). No significant differences were observed between B‐ALL and T‐ALL groups with respect to the proportions of T‐helper (CD4^+^CD8^−^) and cytotoxic T cells (CD8^+^CD4^−^).

Analysis of Treg subsets revealed notable differences between disease subtypes and remission status. The T‐ALL‐BR group exhibited significantly higher frequencies of classical Tregs compared with the remission group (*p* = 0.04). In contrast, naïve Treg cells (CD25^++^CD45RA^+^) were significantly increased in the T‐ALL‐AR group relative to T‐ALL‐BR patients (*p* = 0.01), while low‐expression Treg cells (CD25^+^CD45RA^−^) were significantly reduced AR (*p* = 0.04). Within the B‐ALL cohort, the non‐regulatory CD25^−^ CD45RA^−^ subset was significantly higher in B‐ALL‐AR patients than in B‐ALL‐BR patients (*p* = 0.001).

Finally, analysis of T‐cell activation markers showed that CD3^+^CD69^+^ expression increased significantly AR in both B‐ALL and T‐ALL groups (*p* = 0.03 and *p* = 0.04, respectively). Similarly, CD8^+^CD69^+^ T cells were significantly elevated in B‐ALL‐AR patients compared with B‐ALL‐BR patients (*p* = 0.03), indicating enhanced cytotoxic T‐cell activation following remission.

## 4. Discussion

This study provides a comprehensive immunophenotypic evaluation of peripheral lymphocyte subsets in children and adolescents with ALL, including the major subtypes B‐ALL and T‐ALL. Immune profiles were examined at diagnosis and AR and compared with those of age‐ and sex‐matched HCs. The findings demonstrate substantial immune dysregulation at diagnosis, characterised by reduced TCR expression, altered lymphocyte subset distribution, impaired lymphocyte activation and expansion of Treg populations.

Through stage‐specific immunophenotypic analysis conducted before and AR, the present study provides additional insights into immune dysregulation in paediatric ALL. By evaluating Treg subsets together with effector T cells, NK/NKT populations, and early activation markers, this work expands the current understanding of the immune landscape associated with disease progression and treatment response. Previous investigations have reported alterations in Treg frequency and function in ALL and their association with disease burden and therapeutic outcomes, highlighting their immunosuppressive role within the leukaemia microenvironment [19, 21]. Although partial immune recovery was observed following remission, several abnormalities persisted, suggesting incomplete immunological restoration after treatment [22].

Children with newly diagnosed ALL showed significant suppression of both TCR αβ^+^ and γδ^+^ T‐cell subsets [23, 24]. This reduction is consistent with mechanisms of tumour‐mediated immune evasion, whereby malignant blasts interfere with T‐cell priming through downregulation of antigen presentation pathways or secretion of inhibitory cytokines [25]. Similar alterations have been described in other haematological malignancies, where impaired TCR signalling contributes to functional exhaustion and defective antigen recognition [26, 27]. The high blast burden present at diagnosis may partly explain the immune dysregulation observed in the ALL‐BR group, particularly the reduction in TCR expression and alterations in T‐cell subsets. In addition, the decreased frequency of CD3^+^CD69^+^ T cells supports the concept of impaired early T‐cell activation in active diseases [28, 29]. The concurrent reduction of CD4^+^ T‐helper cells together with the expansion of CD8^+^ cytotoxic T cells suggests a skewed immune response that may not effectively control tumour progression. Although cytotoxic T cells were numerically increased, their functional competence may be limited in the context of reduced activation signalling [30, 31].

A prominent finding of the present study was the expansion of Treg subsets, particularly classical Tregs and memory‐like Tregs. These cells possess strong immunosuppressive properties and are known to inhibit effector T‐cell responses, thereby facilitating tumour immune escape. Increased Treg frequencies have been reported previously in paediatric ALL and have been associated with the disease burden and treatment response [19]. In the present cohort, expansion of memory Tregs (CD25^+++^ CD45RA^−^) together with reduction of CD25^−^ CD4^+^ and CD25^−^ CD45RA^−^ populations suggests a shift from naïve and resting effector T cells towards an immunoregulatory phenotype [22, 32]. Although Treg frequencies decreased following remission, residual abnormalities indicate that immune recovery may lag behind clinical remission. Similar observations have been reported in paediatric cohorts, where Treg modulation persists during immune reconstitution after chemotherapy [33, 34].

Beyond T‐cell alterations, changes were also observed in innate lymphocyte populations. The proportion of NKT cells (CD3^+^ CD56^+^) was increased in both ALL‐BR and ALL‐AR groups, possibly reflecting a compensatory immune response to impaired adaptive immunity. Although the precise role of NKT cells in ALL remains incompletely understood, their expansion may represent an attempt to maintain immune surveillance in the presence of leukaemic cells. In contrast, CD56^bright^ NK cells were significantly reduced in patients following remission. These NK cells are typically involved in cytokine production and immunoregulation, and their depletion may result from treatment‐related lymphoid toxicity or altered NK‐cell maturation pathways. Such changes may contribute to impaired immune regulation and reduced surveillance against MRD. These findings support the concept that chemotherapy‐associated immune remodelling may alter NK‐cell maturation and regulatory function during immune reconstitution.

Evidence of partial immune reconstitution was observed in patients who had achieved remission. The expression of TCR αβ^+^ and γδ^+^ subsets returned to levels comparable to those of HCs, and total CD3^+^ lymphocyte frequencies increased. In addition, CD4^+^ CD69^+^ expression was elevated in the remission group, suggesting the recovery of helper T‐cell activation. However, persistent elevation of CD8^+^ T cells and continued reduction of CD56^bright^ NK cells indicate that immune recovery remains incomplete. These findings highlight the complex dynamics of immune restoration in paediatric ALL, where the numerical recovery of lymphocyte populations may not fully reflect functional immune competence. Monitoring immune subset dynamics during and after treatment may therefore provide additional insights into relapse risk and long‐term immune competence.

Subtype‐stratified analyses suggested differences between B‐ALL and T‐ALL patients. B‐ALL and T‐ALL arise from distinct developmental lineages and may therefore exhibit different patterns of immune dysregulation. In the present study, patients with B‐ALL showed greater recovery of TCR αβ^+^ T cells AR and more pronounced depletion of B‐cell populations than those with T‐ALL, which may reflect the direct impact of B‐lineage malignancy on the non‐malignant B‐cell compartment. In contrast, T‐ALL patients showed a higher CD8^+^CD69^+^ expression at diagnosis, which may indicate a more pronounced, although potentially ineffective, cytotoxic response. Differences were also observed in Treg dynamics across remission states. Although the study was not powered for definitive subtype‐specific conclusions, these observations suggest that patterns of immune dysregulation may differ by disease lineage and highlight the importance of considering the ALL subtype when interpreting immune profiles.

The immunological alterations identified in this study have potential clinical implications. Expansion of suppressive Treg cells together with impaired early T‐cell activation may contribute to tumour immune escape and could serve as biomarkers of disease activity or treatment response. Moreover, the persistence of immune abnormalities AR suggests that immune monitoring may remain relevant beyond the induction phase of therapy. Therapeutic strategies targeting regulatory immune pathways, including Treg modulation or enhancement of NK‐cell function, may therefore represent promising adjuncts to conventional chemotherapy in paediatric ALL.

## 5. Limitations and Future Directions

While this study provides important insights into immune dysregulation in paediatric ALL, several limitations should be acknowledged. First, the cross‐sectional design precludes the evaluation of temporal immune changes throughout the course of treatment. Second, the relatively modest cohort size may limit the generalisability of the findings and reduce the statistical power for detecting subtle subgroup differences. Although subtype analyses were performed, the sample size of the T‐ALL subgroup was limited, which may restrict the strength of conclusions regarding lineage‐specific immune alterations. Accordingly, subtype‐specific findings should be interpreted with caution and considered hypothesis‐generating.

In addition, intracellular FOXP3 staining could not be performed due to limitations in available fluorochrome combinations within the applied flow cytometry panel. Consequently, CD25 and CD45RA expressions were used as surrogate markers for identifying Treg subsets. Although these markers have been widely used for phenotypic identification of Treg cells, future studies incorporating multiparametric flow cytometry and functional assays would allow more precise characterisation of Treg suppressive activity.

Finally, the functional properties of NKT cells and the mechanisms underlying the depletion of CD56^bright^ NK cells were not assessed in the present study and warrant further investigation in larger longitudinal cohorts.

## 6. Conclusion

This study provides a comprehensive immunophenotypic characterisation of peripheral blood lymphocyte subsets in children and adolescents with ALL at diagnosis and following remission. The findings demonstrate that untreated ALL is associated with substantial immune dysregulation, characterised by reduced TCR expression, impaired T‐cell activation, expansion of Treg subsets, and alterations in NK and NKT‐cell populations. Together, these changes indicate a profoundly immunosuppressive environment that may facilitate disease progression.

Although several immune parameters improved following remission, residual abnormalities persisted, including sustained elevation of cytotoxic T cells and depletion of CD56^bright^ NK cells. These findings suggest that immune recovery following therapy may remain incomplete and may contribute to impaired immune surveillance and increased vulnerability to relapse.

The subtype‐stratified findings should be interpreted cautiously but suggest that lineage‐associated immune differences warrant further investigation in larger cohorts. Strategies aimed at modulating Treg activity or restoring NK‐cell function may enhance anti‐leukaemic immunity. Future longitudinal and mechanistic studies are required to clarify the dynamics of immune reconstitution and to identify immunological biomarkers that may improve risk stratification and therapeutic outcomes in paediatric ALL.

**Nomenclature**
ALL: Acute lymphoblastic leukaemia
ALL‐AR: Acute lymphoblastic leukaemia after remission
ALL‐BR: Acute lymphoblastic leukaemia before remission
BMI: Body mass index
CBC: Complete blood count
HC: Healthy controls
IQR: Interquartile range
NK: Natural killer cells
NKT: Natural killer T cells
PBMC: Peripheral blood mononuclear cells
PLR: Platelet‐to‐lymphocyte ratio
TCR: T‐cell receptor
Treg: Regulatory T cells
WBC: White blood cells
APC: Allophycocyanin
CD4^+^: T‐helper cells
CD8^+^: Cytotoxic T cells
FACS: Fluorescence‐activated cell sorting
FITC: Fluorescein isothiocyanate
PerCP: Peridinin‐chlorophyll‐protein.

## Author Contributions

Concept and study design: Hanaa Nafady‐Hego, Hanan M. Abd Elmoneim, Talat Albukhari, Arwa Flemban and Asmaa Nafady. Drafting of the manuscript: Hanaa Nafady‐Hego, Hanan M. Abd Elmoneim, Arwa Flemban, Saeed Kabrah, Ruba Almaghrabi and Samar N. Ekram. Data acquisition: Hanaa Nafady‐Hego, Hanan M. Abd Elmoneim, Talat Albukhari, Arwa Flemban, Hams Alaa, Asmaa Nafady and Enas Aly. Data analysis: Hanaa Nafady‐Hego, Hams Alaa, Saeed Kabrah and Samar N. Ekram. Data interpretation: Hanaa Nafady‐Hego, Hanan M. Abd Elmoneim, Talat Albukhari, Arwa Flemban, Asmaa Nafady, Saeed Kabrah, Ruba Almaghrabi, Enas Aly and Samar N. Ekram. Critical appraisal and review: Hanaa Nafady‐Hego, Hanan M. Abd Elmoneim, Talat Albukhari, Arwa Flemban, Saeed Kabrah, Ruba Almaghrabi and Samar N. Ekram.

## Funding

This research did not receive any specific grant from funding agencies in the public, commercial or not‐for‐profit sectors.

## Ethics Statement

The study was conducted in accordance with the most recent Helsinki Declaration and received ethical approval from the Faculty of Medicine, Qena University (Study Number: SVU_MED_CCP031_4_22_11_48). Informed consent was obtained from patients and healthy controls, and parental authorisation was provided.

## Conflicts of Interest

The authors declare no conflicts of interest.

## Supporting Information

Additional supporting information can be found online in the Supporting Information section.

## Supporting information


**Supporting Information** Table S1: Demographic, haematological, biochemical and coagulation parameters of paediatric patients with acute lymphoblastic leukaemia before remission (ALL‐BR), after remission (ALL‐AR) and healthy controls (HC). Figure S1: Flow cytometric gating strategy for identification of lymphocyte subsets, NK/NKT‐cells and regulatory T‐cells in peripheral blood mononuclear cells.

## Data Availability

The data that support the findings of this study are available from the corresponding author upon reasonable request.
