# Supplementary material for: Immunophenotypic Profiling of Lymphocyte Subsets in Children and Adolescents With Acute Lymphoblastic Leukaemia: Immune Dysregulation Before and After Remission
Source: J Immunol Res. 2026 Jul 2;2026:5591215. doi: 10.1155/jimr/5591215 (PMC13328831; doi:10.1155/jimr/5591215)
Supplement: Supplementary file 1 — Supporting Information Table S1: Demographic, haematological, biochemical and coagulation parameters of paediatric patients with acute lymphoblastic leukaemia before remission (ALL‐BR), after remission (ALL‐AR) and healthy controls (HC). Figure S1: Flow cytometric gating strategy for identification of lymphocyte subsets, NK/NKT‐cells and regulatory T‐cells in peripheral blood mononuclear cells. [file JIMR-2026-5591215-s001.docx]

30^th^ May 2026

Associate Editor, Journal of Immunology Research

Response to the Editor: Manuscript ID **5591215**

Title (revised): **Immunophenotypic Profiling of Lymphocyte Subsets in Children and Adolescents with Acute Lymphoblastic Leukaemia: Immune Dysregulation Before and After Remission**

**Journal:** *Journal of Immunology Research*

**Manuscript Type:** Original Research Article

Dear Editor,

Thank you for your email and for the opportunity to finalise our manuscript for publication. We appreciate your careful review of the accepted paper. We have addressed all requested points in the revised submission files as follows:

1. **Affiliation format**

All author affiliations have been revised in both the submission system and the manuscript file to follow the required order: (1) department, (2) institution, and (3) city/country. The corresponding author’s affiliation has also been standardised. The affiliation list appears on the title page of the manuscript.

2. **Editable high-resolution figures**

Editable versions of all figures have been prepared and uploaded separately under the Figure file section. The manuscript contains five figures, which have now been provided as separate editable files to facilitate journal style adjustments to lines, fonts, and other graphical elements.

3. **Supplementary material descriptions**

We have added concise descriptions of all supplementary material files in the manuscript file immediately before the Reference section. This corresponds to the supplementary items cited in the manuscript, including Supplementary Table S1 and Supplementary Figure S1.

4. **Removal of tracked supplementary file**

The file “Leukemia manuscript Supplementary section tracked.docx” has been removed from the supplementary section, as requested.

We trust that the revised files now meet the journal’s production requirements. Please let us know if any additional modifications are needed.

Thank you for your time and consideration.

Sincerely,

**Arwa F. Flemban, PhD**
Corresponding author
Department of Pathology, Faculty of Medicine
Umm Al-Qura University, Makkah, Saudi Arabia
E-mail: [aflemban@uqu.edu.sa](mailto:aflemban@uqu.edu.sa)

(on behalf of all co-authors)
